# Detection of mutations in *SF3B1*, *EIF1AX* and *GNAQ* in primary orbital melanoma by candidate gene analysis

**DOI:** 10.1186/s12885-018-5190-z

**Published:** 2018-12-17

**Authors:** Anna M. Rose, Rong Luo, Utsav K. Radia, Helen Kalirai, Sophie Thornton, Philip J. Luthert, Channa N. Jayasena, David H. Verity, Sarah E. Coupland, Geoffrey E. Rose

**Affiliations:** 1 Orbital Service, Moorfields Eye Hospital, City Road, London, EC1V 2PD UK; 2 UCL Institute of Ophthalmology, London, UK; 3 Department of Medicine, Imperial College, London, UK; 4 Department of Molecular and Clinical Cancer Medicine, University of Liverpool, Liverpool, UK; 5 Department of Cellular Pathology, Royal Liverpool University Hospital, Liverpool, UK

**Keywords:** Orbit, Melanoma, Primary, SF3B1, EIF1AX

## Abstract

**Background:**

Ocular melanoma is a rare but often deadly malignancy that arises in the uvea (commonest primary site), conjunctiva or the orbit. Primary orbital melanoma (POM) is exceedingly rare, with approximately 60 cases reported to date. Despite recent advances in our understanding of the genetics of primary uveal and conjunctival melanomas, this information is lacking for POM.

**Methods:**

DNA was extracted from 12 POM tissues, with matched germline DNA (where available). MLPA was conducted to detect chromosomal alterations and Sanger sequencing used to identify point mutations in candidate melanoma driver genes (*BRAF, NRAS, KRAS, GNA11, GNAQ*), and other genes implicated in melanoma prognosis (*EIF1AX, SF3B1*). Immunohistochemistry was performed to analyse BAP1 nuclear expression.

**Results:**

MLPA detected copy number alterations in chromosomes 1p, 3, 6 and 8. Sequencing of melanoma driver genes revealed *GNAQ* (p.Q209L) mutations in two samples; although it is possible that these samples represent extraocular spread of an occult uveal melanoma. A recurrent mutation in *SF3B1* (p.R625H) was observed in indolent, but not aggressive, tumours; a mutation in *EIF1AX* (p.N4S) was detected in one patient with non-aggressive disease.

**Conclusions:**

*EIF1AX* and *SF3B1* mutations appear have a role in determining the clinical course of POM and detection of these changes could have clinical significance. Further in depth analysis of this rare group using differing ‘omic technologies will provide novel insights into tumour pathogenesis.

## Background

Ocular melanoma is a rare, generally lethal, malignancy that can arise in the uveal tract, the conjunctiva, or the ocular adnexa (eyelid or orbit). Orbital melanoma occurs either as primary disease, as secondary disease (local invasion from an ocular or sinonasal primary tumour), or as a metastasis from the contralateral eye or from the skin. Melanoma accounts for 5–20% of metastatic and secondary orbital malignancies, but only a very small proportion of primary orbital neoplasia [1–3]. Primary orbital melanoma (POM) is extremely rare, with approximately 60 cases reported to date. It is possible that POM arise from melanocytic cells lining the leptomeninges or ciliary nerves, or from ectopic intraorbital nests of melanocytes [4]. POM can occur de novo, but it is sometimes reported in association with pigmentary changes of the periocular tissues – such as naevus of Ota, blue cellular naevus or oculo-dermal melanosis [5]. The prognosis of POM appears to be quite variable. Generally, the disease is considered to have a very poor prognosis, but there appears to be a subset of patients who have long-survival [6, 7].

The genetic aberrations that drive cutaneous melanoma (CM) and uveal melanoma (UM) are well-described and quite distinct from each other. Mutations in the RAS pathway are found in > 75% of CM and have also been described in conjunctival melanoma, whereas mutations in *GNAQ* and *GNA11* are found in ~ 85% of UM [8, 9]. Several genes have also recently been implicated in UM prognosis, such as *BAP1*, *SF3B1* and *EIF1AX*. There are no data, however, describing the genetic alterations found in POM. In this study, we examined POM for alterations in candidate melanoma driver genes (*BRAF, NRAS, KRAS, GNA11, GNAQ*) and genes implicated in UM prognosis (*EIF1AX, SF3B1, BAP1*).

## Methods

This study received ethics approval from Moorfields Eye Hospital Biobank ethics board (15/SW/0104). Informed, written consent was obtained from patients and research adhered to the tenets of the Declaration of Helsinki. Twelve tumour DNA samples from 11 patients were included in the study; the demographics and clinical course (including disease presentation, occurrence of metastasis and overall survival) of the patients has been previously published but is also summarised in Table 1 [7]. Prognosis was defined as poor (survival of < 6 months with or without metastasis), intermediate (survival > 6 months with local or systemic spread of disease) or good (survival > 6 months without spread; or very late recurrence of disease (> 10 years)).

**Table 1:** Summary of clinical characteristics of 11 patients with primary orbital melanoma. “RT” denotes fractionated external beam radiotherapy. † indicates a deceased patient

Case no.	Gender	Age at onset (years)	Side	Primary treatment of orbital disease	Orbital progression	Time orbital treatment to orbital recurrence (months)	Systemic progression	Time orbit to systemic disease (months)	Systemic therapy	Survival (months)	Notes
1	F	81	R	Debulking	N	–	Y	0		3†	Too unwell for adjuvant radiotherapy
2	M	40	L	Exenteration	N	–	Y	0		4†	Too unwell for adjuvant radiotherapy
4	F	58	L	Exenteration	Unknown	–	Unknown	–		25	Patient declined adjuvant radiotherapy & active follow up
5	M	45	L	Debulking + RT	Y	6	N	–		37	Exenteration after orbital progression
6	F	84	R	Exenteration + RT	N	–	Y	12	Palliative RT for bone metastases	18†	Conjunctival melanosis
8	M	45	L	Debulking + RT	Y	7	Y	45	Liver resection	91	Nevus of Ota Exenteration after orbital progression
9	F	47	L	Debulking + RT	Y	161	Y	168	Nil active	174†	Conjunctival nevus Late exenteration
10	M	46	R	Debulking +RT	N	–	N	–		26	
11	F	43	L	Debulking + RT	N	–	N	–		188	West African
12	F	70	L	Debulking +RT	N	–	N	–		22	
13	M	55	R	Debulking + RT	Y	2	Y	5	Immunotherapy	35	Immunotherapy for progressive systemic and orbital disease

### DNA extraction

Tumour DNA was extracted from surgical samples as previously described [10]. Germline DNA was extracted from peripheral blood using standard phenol-based method.

### MLPA

MLPA was undertaken using the SALSA MLPA P027-C1 kit (MRC Holland, The Netherlands) according to manufacturer’s instructions and previously published methods [10], to detect copy number alterations of chromosomes 1p, 3, 6 and 8. Classification of chromosomes was based on the dosage quotients generated and ≥ 75% probes on a particular chromosome being classified as loss, normal or gain as previously described [11]. Chromosomes were considered ‘unclassified’ when the criteria could not be met.

### Sanger sequencing

PCR was performed using custom primer sequences to target regions (Table 2) using PCR Master Mix 2X, ThermoFisher Scientific UK (cycle conditions available upon request). Sanger sequencing was performed using BigDye Terminator v3.1 on ABI3730 using a standard protocol (both Applied Biosystems, UK). Sequence data was analysed on Seqman Pro (DNASTAR, USA). Identified changes were validated in a second PCR and sequencing reaction. Validated mutations were assayed in germline genomic DNA from the patient, to confirm that they were a somatic mutation.

**Table 2:** Regions of interest assayed and primer sequences used. The commonly reported mutations in the exons of interest were taken from individual gene reports on COSMIC database [13]

Gene	Exon	Forward primer	Reverse primer	Previously reported mutations	Implicated in
*BRAF*	11	CTGTTTGGCTTGACTTGAC	CATATCCTATTATGACTTG	Minor mutation hotspots at codons 466 and 469	Driver mutation, cutaneous melanoma
	15	CTGATAGGAAAATGAGAT	CAGCAGCATCTCAGGGCC	p.V600E	
*NRAS*	2	GTGGCTCGCCAATTAAC	CCGACAAGTGAGAGAC	Mutation hotspots at codons 12 and 13	Driver mutation, cutaneous melanoma
	3	GCATTGCATTCCCTGTGG	GAACACAAAGATCATCC	p.Q61R, p.Q61L	
*KRAS*	2	GATAGTGTATTAACC	AACCTTTATCTGTATC	Mutation hotspots at codons 12 and 13	Driver mutation,cutaneous melanoma
	3	GTGCACTGTAATAATCT	AATTACTCCTTAATGTC	p.Q61R, p.Q61H	
*GNAQ*	4	CTTTCCGTAGACAGCTTTG	GTACTCAAGGCATAAAAG	p. R183Q	Driver mutation, uveal melanoma
	5	GCTATATTTATGTTGAC	CTATCATTTACTTGTATC	p.Q209L, p.Q209R, p.Q209P	
*GNA11*	4	GCAGCCGGCCTGAGCA	ACACACACTGAGGATG	p. R183C	Driver mutation, uveal melanoma
	5	GCCAGGTGGCTGAGT	GCAGGGCCTTACTGG	p.Q209L, p.Q209P	
*SF3B1*	14	GGCCGAGAGATCATTTCTAAT	AAGAAGGGCAATAAAGAAGGA	Mutation hotspot at codon 625, and multiple other hotspots	Prognosticating mutation in uveal melanoma, possible driver mutation
*EIF1AX*	1	GCCACGCCTGCGTCATAAAG	CGAGCTCAGAGTCGCGTGTG	Mutation hotspots at codons 2,3,4,6	Prognosticating mutation in uveal melanoma, possible driver mutation
	2	AAAGGAAATTCCAAGAAGGGTAGGG	TAATCGTGCCACCACACTTCACC	Mutation hotspots at codons 13, 15	

### BAP1 immunohistochemistry

BAP1 protein expression was examined in 4 μm sections of each POM case using a mouse anti-BAP1 antibody (sc-28,383, Santa Cruz, Insight Biotechnology Ltd., Middlesex, UK) and the DAKO Envision FLEX Kit, according to previously published methods [12]. POM were classified as either BAP1 positive or BAP1 negative according to whether the tumour cells displayed nuclear staining or not, respectively.

## Results

### Copy number changes in chromosomes 6 and 8 are frequently observed in POM

MLPA was performed to investigate chromosomal changes within POM. Copy number alterations were obtained for 7/11 tumour samples (Table 3). Four tumours were reported as disomy 3 and three cases had unclassifiable chromosome 3 status. The most frequent change was gain of chromosome 6p, in 5/7 cases. Polysomy 8q was reported in four cases; gain of chromosome 1p was also observed (2/7 cases). It was not possible to correlate chromosomal alterations with outcome because of the small number of patients for whom data were available.

**Table 3:** MLPA, gene sequencing and BAP1 IHC in eleven primary orbital melanoma cases. The case number refers to the clinical histories previously published [7]. G gain, U unclassified, N normal copy normal, L loss; − reference sequence, + heterozygous mutation observed

Case ID	BAP1 IHC	MLPA result - chromosome						*SF3B1* (R625H)	*EIF1AX* (N4S)	*GNAQ* (Q209L)
		1p	3	6p	6q	8p	8q			
1	Positive	G	U	G	N	N	G	–	–	–
2	Positive	MLPA QC fail						–	–	–
4	Negative	N	N	G	N	N	G	+	–	+
5	Positive	MLPA QC fail						–	–	+
6	NA	N	N	N	N	N	N	–	–	–
8	Positive	N	U	G	N	N	N	–	–	–
9	Positive	G	N	G	L	L	G	+	–	–
10	Positive	N	N	G	N	N	G	–	+	–
11	Positive	U	U	U	G	G	N	+	–	–
12	Positive	MLPA QC fail						+	–	–
13	Negative	MLPA QC fail						–	–	–

### Analysis of driver mutations in POM

**Fig. 1:**
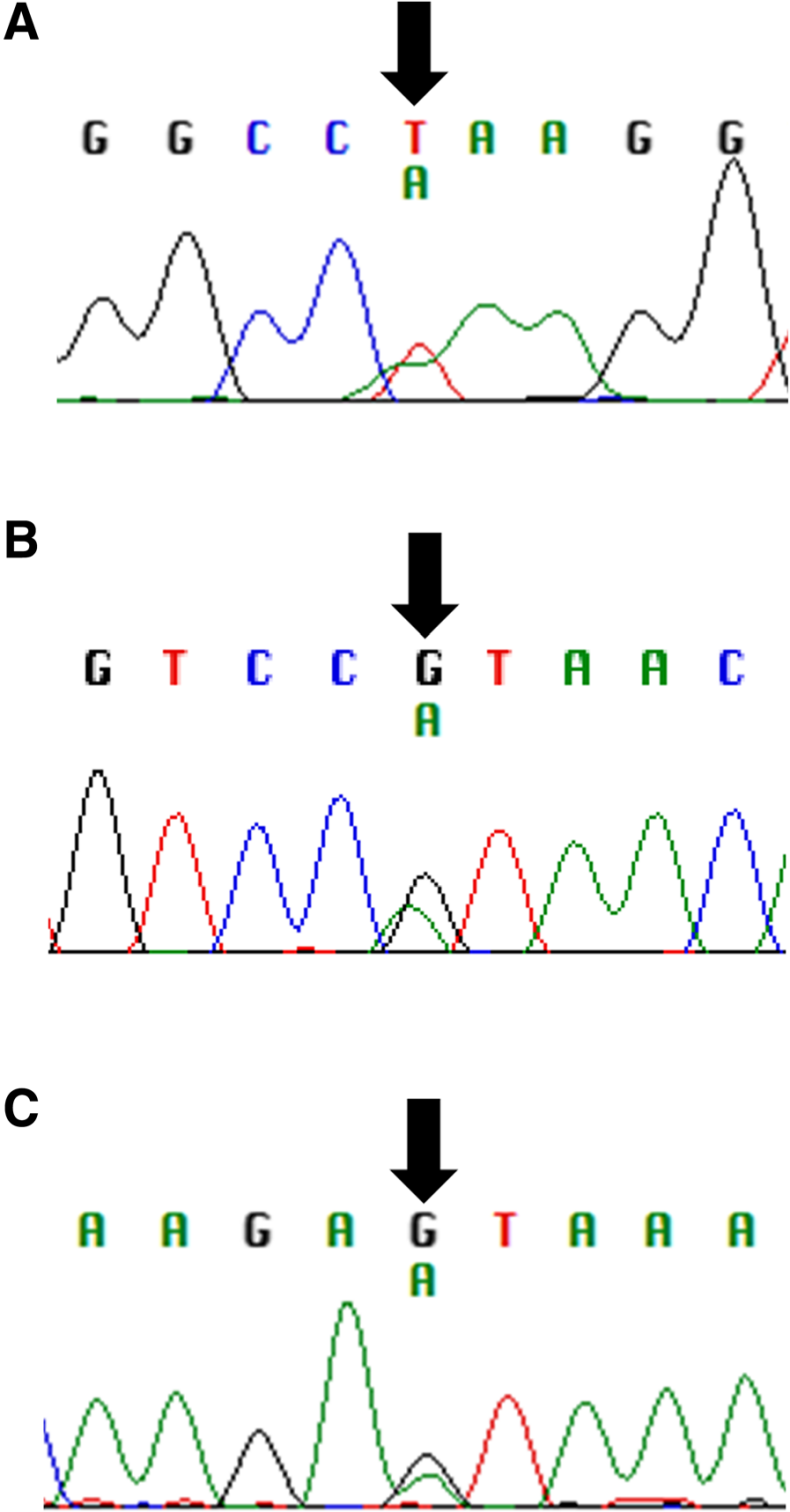
Point mutations identified in tumour tissue of primary orbital melanoma. **a** Two cases (patients 4 and 5) harboured a heterozygous mutation in *GNAQ* (c.A626T, p.Q209L). **b** A recurrent heterozygous mutation in *SF3B1* (c.G1874A, p.R625H) was identified in four patients with favourable prognoses (cases 4, 9, 11, 12). **c** One patient, case 10, carried a heterozygous change in *EIF1AX* (c.A11T, p.N4S)

**Fig. 2:**
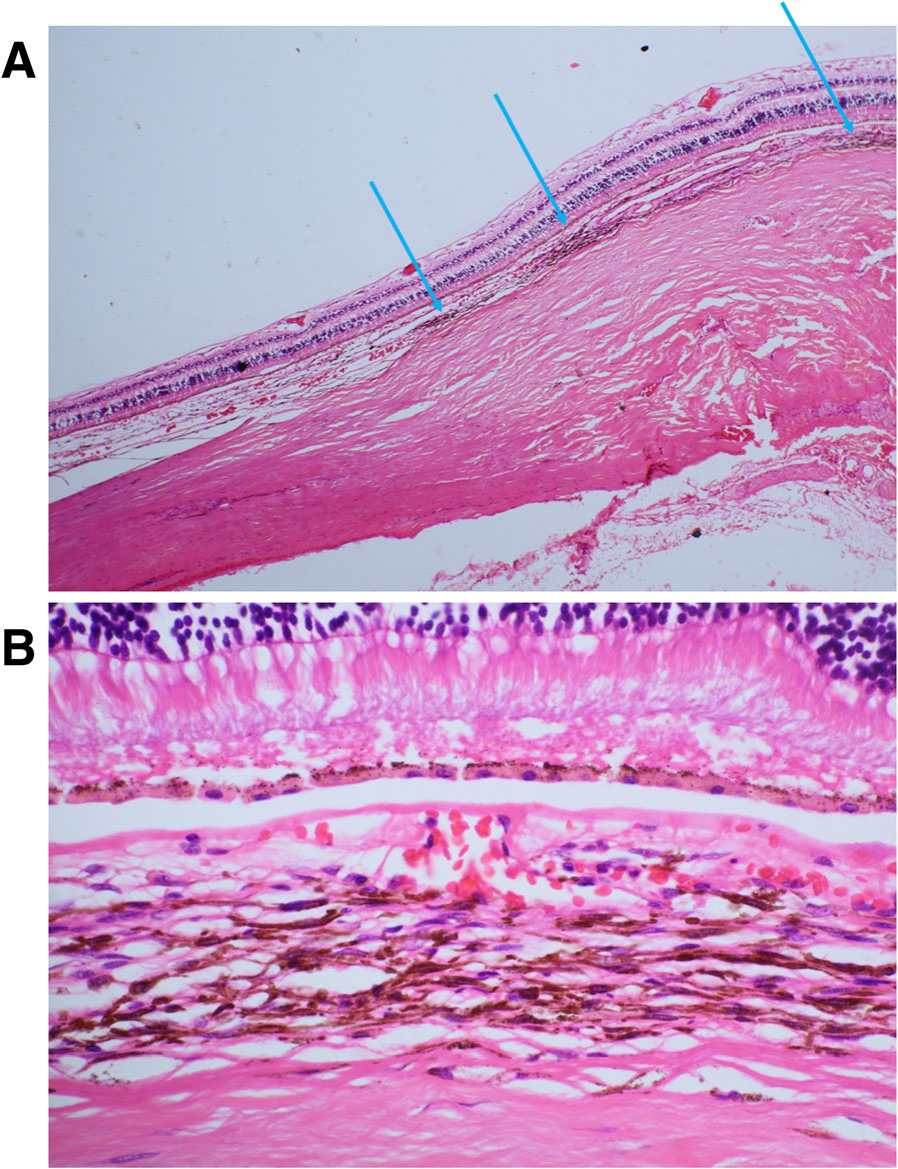
**a** H&E staining of serial slices of exenteration specimen revealed several small regions of increased choroidal melanosis in case 4 (arrowed). **b** Representative image of region with increased pigmentation, showing that the pigmented cells were not malignant in appearance

Sequencing of mutation hotspots in five melanoma driver genes (*BRAF, NRAS, KRAS, GNAQ, GNA11*) did not reveal any changes in 9/11 patients. In two patients (numbers 4 and 5), a change in *GNAQ* was found: c.A626T, p.Q209L (Fig. 1a). *GNAQ* mutations account for approximately 50% of UM driver changes, and p.Q209L is found in 33% of *GNAQ* mutant tumours [13]. It was considered, therefore, whether these two samples could represent an extraocular manifestation of a primary UM, with an occult uveal source. Review of the available imaging did not reveal any possible uveal source. In light of this, the histology slides were re-reviewed by a second expert pathologist (PL, blinded to genetic findings). It was observed that patient 4 had some small regions of increased pigmentation in the choroid that might represent a uveal source of the tumour, although it was felt more likely that these were insignificant findings given the benign appearance and size of the lesions (Fig. 2). In the case of patient 5, the exenteration sample was not available for histological analysis and, so, although there was no evidence of choroidal involvement in the samples examined, it remains impossible to fully exclude an occult uveal source.

### Identification of a recurrent change in *SF3B1*

Sequencing of exon 14 of *SF3B1* revealed that four patients (cases 4, 9, 11, 12) harboured the same mutation; c.G1874A, p.R625H (Fig. 1b). One patient (case 10) was found to harbour a change in exon 1 of *EIF1AX*; c.A11T, p.N4S (Fig. 1c). These changes were not present in the germline DNA of the patients.

It was noted that the patients harbouring the recurrent *SF3B1* mutation all had highly favourable prognosis (Table 4). The mean survival to-date of those patients harbouring the *SF3B1* mutation is 104.8 months (range = 22–188 months), whilst the mean survival of patients without the *SF3B1* mutation is 29.8 months (range = 3–91 months). It is not possible, however, to assess the significance of these differences due to the small sample size. Furthermore, it should be noted that of the seven patients with systemic and/or orbital progression of disease, only one carried the *SF3B1* mutation – and this patient remained without disease progression for over 13 years prior to sudden recurrence, deterioration and death. The *SF3B1* mutation was found in both the first tumour biopsy sample (disease which remained quiescent for 13 years) and the recurrent tumour sample.

**Table 4:** Correlation between prognosis in patients harbouring *SF3B1* and *EIF1AX* changes versus wildtype patients. Deceased patients indicated by †; survival correct to January 2018. The two patients highlighted in grey might represent secondary orbital melanoma with an occult uveal origin

Case no.	Prognosis group	Orbital progression	Systemic progression	Survival (months)	*SF3B1* p.R625H	EIF1AX p.N4S
1	Poor	No	Yes	3†		
2		No	Yes	4†		
13	Intermediate	Yes	Yes	25		
5		Yes	No	37		
6		No	Yes	18†		
8		Yes	Yes	91		
9	Good	Yes (after 13 years)	Yes (after 13 years)	174†	YES	
10		No	No	26		YES
11		No	No	188	YES	
12		No	No	22	YES	
4		Unknown	Unknown	35	YES	

### BAP1 immunohistochemistry

**Fig. 3:**
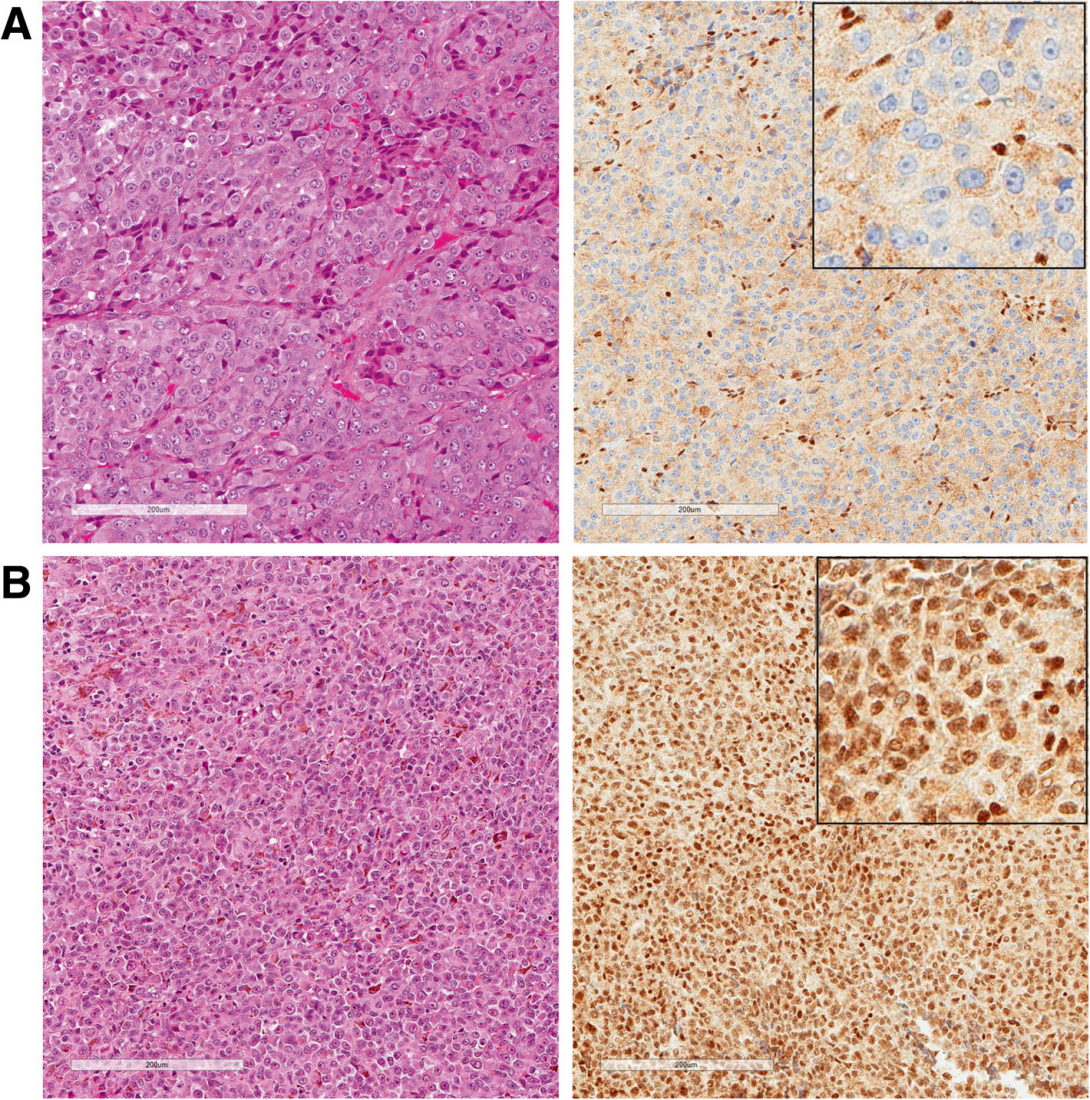
Representative images of BAP1 immunohistochemistry. **a** H&E and nuclear BAP1 (nBAP1) negative tumour (Case ID 13), with normal staining found in admixed lymphocytes. **b** H&E and nBAP1 positive tumour (Case ID 12). Insets show nBAP1 staining at ×40 magnification

BAP1 IHC was possible in 10 cases, a single case had no remaining available tumour material. Nuclear BAP1 (nBAP1) expression was noted in the tumour cells in eight cases and was absent in two (Fig. 3). Of the two nBAP1 negative cases, one showed disomy of chromosome 3 and the presence of *SF3B1* and *GNAQ* mutations, whilst in the second case MLPA had not yielded any results and no mutations were detected.

## Discussion

In this study we examined the largest reported clinical series of POM for mutations implicated in melanoma, giving the first report of genetic alterations in this rare tumour. It was demonstrated that POM appears to be genetically distinct to CM, but shares some overlapping features with UM; however, a distinct driver mutation might exist in at least some cases of POM. Furthermore, mutations in *SF3B1* and *EIF1AX* might influence prognosis. POM is an extremely rare tumour, with approximately 60 cases reported to-date in the literature. One striking feature is the highly variable prognosis – whilst most reported cases have a dismal prognosis, a subset of patients appear to follow a relatively benign course [6, 7]. This feature was certainly observed in the patient cohort used in this study – some patients had aggressive primary disease with widespread systemic involvement and rapid deterioration; whereas other patients survived for more than a decade [7].

POM were not associated with monosomy 3 in the small number of tested samples, which is a frequent chromosomal alteration in UM associated with poor prognosis [14]. In addition, although polysomy 8q was observed in 4/7 POM in this cohort, all were associated with patients who had a good prognosis, which again is contrary to its association with a poor outcome in UM. These data would suggest that chromosomal alterations in POM do not follow a similar pattern to that observed in UM, although testing of more cases will be important. Similarly, loss of nBAP1, which is associated with a poor prognosis and monosomy of chromosome 3 in UM [12, 15, 16] was noted in only two cases and was neither associated with a poor outcome nor with monosomy of chromosome 3.

Mutations in genes of the *MAPK* pathway are known to drive the majority of cases of CM. Mutations in *BRAF* account for the majority of driver mutations (up to 60%) [17]. However, mutations in *NRAS* and *KRAS* are also relatively frequent (13–25 and 2%, respectively) [18]. Screening for mutations in these genes did not, however, reveal any changes in our POM cohort. Mutations in two Gq alpha sub-unit genes, which interact indirectly with the MAPK pathway, are found frequently in UM: *GNAQ* (~ 45%) and *GNA11* (~ 35%) [19, 20]. In our cohort, two cases (patients 4 and 5) were found to carry a heterozygous mutation in *GNAQ* (p.Q209L). This change is the most frequently observed change in *GNAQ* mutant UM, occurring in one third of such cases [21]. This dominant-negative mutation alters the catalytic (GTPase) domain of GNAQ and results in a constitutively active protein [22]. Expression of *GNAQ* p.Q209L in mice resulted in malignant transformation of melanocytes and increased signalling through the MAPK pathway [21]. It is often speculated whether POM cases are true primary disease, or whether an occult uveal source might be present. It was considered necessary, therefore, to assess whether these two cases could be occult uveal tumours, given the frequency of *GNAQ* p.Q209L in UM. The imaging of both cases was reviewed, and a uveal source could not be discerned. The tumour histology of the cases was re-reviewed by an expert pathologist, without knowledge of the genetic findings. In one case harbouring the *GNAQ* change, there was evidence of very minor choroidal pigmentary changes which could represent a possible uveal source; in the other case, there was insufficient biopsy material to fully exclude a uveal source. It is possible, therefore, that these two cases might indeed have an occult uveal source. It is also plausible, however, that these are POM that share common genetic features with UM. Mutations in other G protein genes, such as *CYSLTR2* and *PLCB4* have been reported in a small number of UM patients and these could be sequenced in our cohort to extend the study further [23, 24].

Next, it was considered whether mutations in *SF3B1* or *EIF1AX* contributed to the pathogenesis of disease and/or variable prognosis seen within our cohort. Mutually exclusive changes in both genes were found (which might associate with good prognosis), highlighting another feature that overlaps with the genetic landscape of UM. Sequencing of exon 14 of *SF3B1* revealed a recurrent heterozygous mutation (c.G1874A, p.R625H) in four patients – cases 4, 9, 11 and 12. It was noted that all four of these patients had a favourable outcome, with a mean survival to-date of 104.8 months (22–188 months). Three of these patients have not had any local or systemic progression or recurrence of disease. One patient – case 9 – harbouring the *SF3B1* mutation had systemic progression and has since died, however, this was after a remarkable disease-free period of 13 years. The presence of the *SF3B1* mutation was confirmed in both the primary and recurrent tumour of this individual. In contrast, no patients with poor survival, or early local/systemic progression, carried the *SF3B1* change. This would suggest that the observed mutation in *SF3B1* confers a favourable prognosis in POM.

*SF3B1* encodes splicing factor 3b, subunit 1 protein, which is a component of the splicing factor 3b protein complex. This complex is part of the spliceosome, the macromolecular structure responsible for transcriptional mRNA processing. Mutations in *SF3B1* have been implicated as a common driver mutation in myelodysplatic syndromes (MDS), myelofibrosis and chronic myeloid leukaemia [25, 26]. In MDS, *SF3B1* mutations have been associated with favourable overall survival and a lower likelihood of transformation into acute leukaemia [27, 28]. More recently, mutations in *SF3B1* (particularly at codon 625) have been identified in various pigmented tumours, including UM [29], mucosal melanoma [30], leptomeningeal melanoma [31] and blue naevi-like cutaneous melanoma [32]. They are, however, rare in CM [33, 34]. As with MDS, *SF3B1* mutations confer a favourable prognosis in UM, with lower age of onset and concurrent disomy 3 [35]. However, it should be noted that *SF3B1* mutant UM are reported to give rise to late metastasis (median 8.2 years after initial diagnosis) [36]. Furthermore, The Cancer Genome Atlas project reported that UM cases with *SF3B1* mutations have an intermediate prognosis [9]. One of our patients harbouring the change (case 9) did indeed have late onset metastasis to the brain (13 years after diagnosis). It is also interesting to note that secondary melanoma within the orbit was noted to have frequent late recurrence, and the incidence of *SF3B1* in these tumours could be studied [37].

One patient, case 10, was found to harbour a heterozygous mutation in exon 1 of *EIF1AX* (c.A11T, p.N4S). This patient also had a highly favourable prognosis. *EIF1AX*, located on the X chromosome, encodes the eukaryotic translation initiation factor 1A protein. This factor is essential in the initiation phase of translation, through interaction with tRNA and the ribosome [38]. Mutations in exon 1 or 2 of this gene have been frequently reported in UM [39], but also rarer melanoma types including blue-nevus associated melanoma and leptomeningeal melanoma [33, 34]. *EIF1AX* mutations are associated with good prognosis disomy 3 UM, and usually occur in non-metastatic cases [39–41]. This is in agreement with the patient reported here, who displayed all of these features to-date with follow up of over 2 years. It is highly plausible, therefore, that mutations in *EIF1AX* also correspond to good prognosis in POM.

It must be considered whether the changes seen in *SF3B1* and *EIF1AX* are the driver mutation of POM, or a secondary change in the tumour. It is thought that mutations in *SF3B1* arise as a second genetic change in UM and blue nevus-like melanoma, after initial mutation in *GNAQ* or *GNA11*; however, *SF3B1* changes can be driver mutations in MDS [25, 34]. Indeed, in UM, mutations in either gene (*SF3B1* or *EIF1AX*) are almost never seen in the absence of a *GNAQ* or *GNA11* mutation [13]. Whole exome or whole genome sequencing of these tumours will be necessary to elucidate the full spectrum of genetic mutations in POM. A further consideration is the role of underlying precursor or premalignant lesions in the genetic aetiology of POM. The tumour can arise following malignant transformation of pigmentary changes of the periocular tissues – such as naevus of Ota, blue cellular naevus or oculo-dermal melanosis [5]. This was the case in three individuals studied here (Cases 6, 8 and 9; in addition, minor increased choroidal pigmentation was observed on re-examination of Case 4. It would be interesting to study the genetic aberrations in the premalignant tissue, as this might shed light on the temporal occurrence of sequential mutations and their role in pathogenesis.

## Conclusions

In summary, we have provided the first genetic insights into POM. We have shown that – in contrast to CM and UM – mutations in the tested MAPK pathway genes or Gq alpha subunit genes are not implicated in the majority of POM. Furthermore, we have shown that mutations in either *SF3B1* or *EIF1AX* are likely to be associated with a favourable prognosis in POM cases. Testing for these changes in the clinical setting might allow better prognostication for patients. However, it is possible that *SF3B1* mutations are associated with late recurrence of disease, and so long-term follow-up and high index of suspicion should be maintained in all cases of orbital melanoma. Additional ‘omic technologies, such as whole genome sequencing, should be used to explore whether there are other driver mutations involved in the development of these rare tumours.

## Abbreviations

CM: Cutaneous melanoma
IHC: Immunohistochemistry
MAPK: Mitogen-activated protein kinase
MDS: Myelodysplastic syndrome(s)
MLPA: Multiplex ligation dependent probe analysis
PCR: Polymerase chain reaction
POM: Primary orbital melanoma
UM: Uveal melanoma
